# Regulation of Thromboxane Receptor Signaling at Multiple Levels by Oxidative Stress-Induced Stabilization, Relocation and Enhanced Responsiveness

**DOI:** 10.1371/journal.pone.0012798

**Published:** 2010-09-15

**Authors:** Stephen K. Ball, Mark C. Field, John R. Tippins

**Affiliations:** 1 Division of Cell and Molecular Biology, Imperial College, London, United Kingdom; 2 Department of Pathology, University of Cambridge, Cambridge, United Kingdom; University of Illinois at Chicago, United States of America

## Abstract

**Background:**

Thromboxane A_2_ (TxA_2_) is a major, unstable arachidonic acid metabolite, and plays a key role in normal physiology and control of vascular tone. The human thromboxane receptor (TPβ), expressed in COS-7 cells, is located predominantly in the endoplasmic reticulum (ER). Brief hydrogen peroxide exposure increases the efficiency of translocation of TPβ from the ER into the Golgi complex, inducing maturation and stabilization of TPβ. However, the ultimate fate of this post-ER TPβ pool is not known, nor is its capacity to initiate signal transduction. Here we specifically assessed if functional TPβ was transported to the plasma membrane following H_2_O_2_ exposure.

**Results:**

We demonstrate, by biotinylation and confocal microscopy, that exposure to H_2_O_2_ results in rapid delivery of a cohort of TPβ to the cell surface, which is stable for at least eight hours. Surface delivery is brefeldin A-sensitive, indicating that translocation of this receptor cohort is from internal pools and via the Golgi complex. H_2_O_2_ treatment results in potentiation of the increase to intracellular calcium concentrations in response to TPβ agonists U46619 and 8-iso PGF_2α_ and also in the loss of ligand-dependent receptor internalization. Further there is increased responsiveness to a second application of the agonist. Finally we demonstrate that the effect of H_2_O_2_ on stimulating surface delivery is shared with the FP prostanoid receptor but not the EP3 or EP4 receptors.

**Conclusions/Significance:**

In summary, brief exposure to H_2_O_2_ results in an immediate and sustained increase in the surface pool of thromboxane receptor that is capable of mediating a persistent hyper-responsiveness of the cell and suggests a highly sophisticated mechanism for rapidly regulating thromboxane signaling.

## Introduction

Oxidative stress is a common factor in many aspects of cardiovascular disease [1]. Specifically, reactive oxygen species (ROS) impair vascular relaxation and promote apoptosis of endothelial cells, augment expression of adhesion molecules and also lead to the proliferation, hypertrophy and migration of smooth muscle cells, which contribute to development of hypertension and atherosclerosis [2].

Thromboxane A_2_ (TxA_2_) is a major, unstable arachidonic acid metabolite, and plays a key role in normal physiology [3] but is additionally implicated in many pathological states such as unstable coronary artery disease and severe unstable angina [4]. TxA_2_ is an agonist for the G protein-coupled thromboxane receptor, of which two variants, TPα (343 amino acids) and TPβ (407 amino acids), arise by alternate splicing of transcripts derived from a single gene [5] and exhibit distinct tissue expression profiles. There are clear differences between the mechanisms of downstream signaling for these two isoforms. Both TPα and TPβ are coupled to downstream signaling pathways via interaction with predominantly Gq11, and exhibit complex interactions, including activation of protein kinase C, RhoA [6] and AMP-activated protein kinase [7] and can stimulate release of intracellular calcium stores. Regulation of TP signaling itself is similarly complex, with multiple kinase-mediated pathways implicated in receptor desensitization, while oligomerization of the distinct TP splice variant products has also been implicated in modulating function [8], [9].

Here we examined the effects of oxidative stress on the thromboxane receptor to understand the molecular and cellular consequences of exposure to ROS on thromboxane receptor activity. Previously, using COS-7 cells that naturally express TP receptor, we demonstrated that brief exposure to hydrogen peroxide, as a model for acute oxidative stress, significantly increased translocation of TPβ from the ER to the Golgi complex [10]. Translocation of TPα from the ER to the Golgi complex as a consequence of TP activation via a reactive oxygen species-dependent mechanism has also recently been reported [11]. TPβ is rapidly turned over in the ER, while TPβ degradation is modulated following exposure to hydrogen peroxide and was most likely due to activation of the unfolded protein response (UPR), resulting in increased ER folding efficiency of TPβ, and subsequent exit from the ER, effectively removing the polypeptide from the ER-associated degradation system [10]. The TPα splice variant, despite differential tissue expression and signalling properties, undergoes a similar translocation and stabilization essentially indistinguishable from the β-splice variant. Further, four arginine residues between the C-terminal-most trans-membrane region and the C-terminus of TPβ are required for responsiveness to hydrogen peroxide [12]. From these data it appeared that only a very limited population of thromboxane receptors existed on the plasma membrane of COS-7 and human coronary artery smooth muscle cells in the resting state. However, the role of the surface pool, and its capacity to initiate signal transduction were not investigated. The thromboxane receptor signals from the plasma membrane and therefore an understanding of the effects of oxidative stress on the cell surface population and its ability to mediate intracellular events is important.

The present study aimed specifically to determine if TPβ was efficiently transported to the plasma membrane following exposure to hydrogen peroxide. Further, we wished to determine the rate of translocation of TPβ to the cell surface, the stability, fate and functional competence of this pool and, most importantly, if the surface pool was capable of transducing a signal delivered by TP receptor agonists. Finally we also aimed to establish if hydrogen peroxide-stimulated trafficking of TP was specific or mirrored in the behaviour of additional prostanoid receptors. We demonstrate that oxidative stress results in rapid delivery of a large cohort of TPβ to the cell surface, that this cohort of TPβ is both highly stable and capable of mediating enhanced prostanoid responses and that not all prostanoid receptors respond to hydrogen peroxide by increased presence at the plasma membrane.

## Materials and Methods

### Materials

HEK293 and PC3 cells were kind gifts from David Guiliano and Charlotte Bevan (Imperial College, London, UK). The mammalian expression vector pcDNA3.1, Dulbecco's-modified Eagle's medium (DMEM), foetal calf serum, penicillin/streptomycin solution, Fungizone and trypsin-EDTA were all purchased from Invitrogen. TP receptor polyclonal antibody, which recognizes both TPα and TPβ isoforms, was purchased from Cayman Chemical, Estonia. Mouse anti-HA monoclonal antibody and anti-ribosomal S6 goat polyclonal antibody were purchased from Santa Cruz Chemicals. Mouse monoclonal anti-Na^+^-K^+^ ATPase was from AbCam, Cambridge, UK. Fluorescein-conjugated goat anti-mouse polyclonal antibody and goat anti-mouse horseradish peroxidase conjugate were from Sigma. Cell permeant Fura-Red acetoxymethyl ester was obtained from Invitrogen and the amine reactive EZ-Link Sulfo-NHS-LC-Biotin from Pierce. U44619 and 8-iso PGF_2α_ were obtained from Cayman Chemicals. Unless otherwise stated, all other reagents were from Sigma.

### Generation of epitope-tagged thromboxane receptor

Human TPβ cDNA was amplified by PCR with HindIII and EcoRI restriction sites using the following primers CGAAGCTTATGTGGCCCAACGGCAGT and CGCAGTGAATTCCGCCTGTAATCCC AG (restriction sites underlined, 5′ to 3′). The product of the PCR reaction was subcloned into HA-tagged pcDNA3.1 using HindIII and EcoRI restriction sites. Correct orientation of the insert was confirmed by sequencing.

Stable expression of HA-TPβ in HEK293 cells and PC3 culture. To create a stable HEK293 cell line over-expressing HA-TPβ, cultures were transfected with ScaI linearized pcDNA3.1-HA-TPβ using Escort V reagent, according to the manufacturer's guidelines (Sigma). Following a 24 hour incubation period the cells were re-seeded onto 48 well plates containing DMEM supplemented with G418 (800 µg/ml). The cultures were incubated for three weeks during which time the G418-supplemented medium was replaced every three days. G418 resistant colonies were then expanded into 48 well plates. Routinely, HEK293 cells stably expressing HA-TPβ were maintained in DMEM supplemented with 800 µg/ml G418, 10% serum, 1% penicillin/streptomycin and 25 µg/ml Fungizone in a humidified atmosphere 95% air and 5% CO_2_ at 37°C. Upon reaching ∼80% confluence, the cells where recovered with 0.05% trypsin-EDTA solution and reseeded into 25 cm^2^ flasks at a dilution of 1∶10. This reliably yielded cultures of 60–70% confluence after a 24 hour incubation period. PC3 cells were cultured in phenol red-free DMEM supplemented with 10% FCS at 37°C in 5% CO_2_.

### Confocal microscopy

HEK293 cells stably expressing HA-TPβ or PC3 cells were grown on glass coverslips and fixed with 3.7% paraformaldehyde for 30 minutes at room temperature. Non-specific labelling was blocked by incubation for one hour in blocking buffer (1% (w/v) bovine serum albumin in PBS) at room temperature. All subsequent labelling was carried out in blocking buffer (primary antibody 1∶500 and secondary 1∶250). Cells were viewed under a Leica SP2 upright microscope and the images processed in LCS Light (Leica).

### Cell surface biotinylation

Cell surface expression of TPβ was assayed by the biotinylation of surface proteins using the membrane impermeable, amine-reactive biotin analogue SS-biotin (Pierce). Cultures were cooled on ice and washed three times with ice-cold biotinylation buffer (phosphate buffered saline, 15 mM glucose pH 8.0). Biotinylation was carried out using 0.5 mg/ml biotin in biotinylation buffer for 1 hour on ice. Following one wash with ice-cold biotinylation buffer the biotin was quenched with two washes of quenching buffer (phosphate buffered saline supplemented with 10 mM Tris, 100 mM glycine, pH 8.0). Cell lysis was achieved by addition of RIPA buffer to the monolayers (1% Triton X-100 (v/v), 1% sodium deoxycholate (w/v), 0.1% SDS (w/v), 150 mM NaCl, 5 mM EDTA, in PBS, pH 7.5). Following a 15 minute incubation on ice, the cell lysate was cleared by centrifugation at 14 000 rpm for 10 minutes in an Eppendorf 4417C microcentrifuge. The cleared lysates were then incubated for 1 hour with 20 µl streptavidin beads (Sigma) with end-over-end rolling. The streptavidin beads and the supernatant were separated by brief centrifugation and the beads washed three times in lysis buffer. Proteins in the supernatant were precipitated with 15% (w/v) trichloroacetic acid followed by incubation on ice for an hour. The precipitate was recovered by centrifugation at 14 000 g for an hour in a 4417C microcentifuge (Eppendorf). The resulting pellets were washed with ice-cold 90% acetone to remove excess trichloroacetic acid. The precipitated protein and the streptavidin beads were heated to 95°C for 5 minutes in the presence of SDS-PAGE loading buffer and loaded onto discontinuous 12% SDS-PAGE gels.

### Western blotting

Proteins were transferred onto polyvinylidine fluoride membrane (BioRad, Hercules, California) in transfer buffer (25 mM Tris, 192 mM glycine and 20% (v/v) methanol) at 10 volts overnight. The membrane was subsequently blocked for 1 hour with 5% (w/v) non-fat milk in Tris-buffered saline supplemented with 0.05% (v/v) Tween. Both primary and secondary antibodies were used at a concentration of 1∶5000 for one hour with gentle rocking. Detection was carried out by enhanced chemiluminescence followed by exposure to Amersham Hyperfilm. Blots were quantified in ImageJ 1.34s (NIH, USA). HA-TPβ expression was compared by one-way ANOVA with the Bonferroni post test. A P value <0.05 was taken to be statistically significant.

### Calcium mobilization studies

HEK293 cells stably expressing HA-TPβ were plated onto ibidi 8 well μ-Slides (Thistle Scientific, Glasgow). Measurement of [Ca^2+^]_i_ was performed using a ratiometric method with Fura-Red, a dual excitation probe [13]. Loading was carried out for 30 minutes in loading buffer (20 µM Fura-Red, 0.1% (w/v) pluronic F127, 10 mM glucose, 1% (v/v) foetal calf serum in PBS) followed by two washes. All incubations were carried out at 37°C. In experiments testing the effect of hydrogen peroxide, 10 µM H_2_O_2_ was added with the Fura-Red. At the end of the incubation period agonist (U46619 or 8-iso PGF_2α_) was added, calcium mobilization was visualized under a Zeiss Axiovert 200 widefield microscope (excitation 460 and 490 nm emission 590 nm) and the data processed using Volocity 4 software (Improvision, UK). In some experiments a second administration of agonist was given. In these experiments cells were washed three times with loading buffer and re-incubated in the presence of 10 µM H_2_O_2_. In each experiment the second U46619 concentration was the same as the first, i.e. either 1, 30 or 100 nM. Maximal response (R_max_) was measured by the addition of 0.2% (v/v) Triton X-100 and minimal response (R_min_) by the addition of 2 mM EGTA. The ratio of the fluorescence at 460 and 490 nm is a measure of [Ca^2+^]_i_ assuming the K_d_ of Fura-Red is 222 nM. Sigmoidal concentration-response curves were fitted using Prism 4 (GraphPad Software, Inc). Curves obtained in the presence and absence of hydrogen peroxide were compared by two-way ANOVA with the Bonferroni post test. A P value <0.05 was taken to be statistically significant.

## Results

### Exposure to hydrogen peroxide leads to a rapid and persistent increase in TPβ receptor expression at the cell-surface

HEK293 cells express no detectable amounts of endogenous TPs but have been used as a relevant cellular model for study of TPβ behavior previously [14], [15], [16]; hence we selected this cell line for much of our analysis.

**Figure 1:**
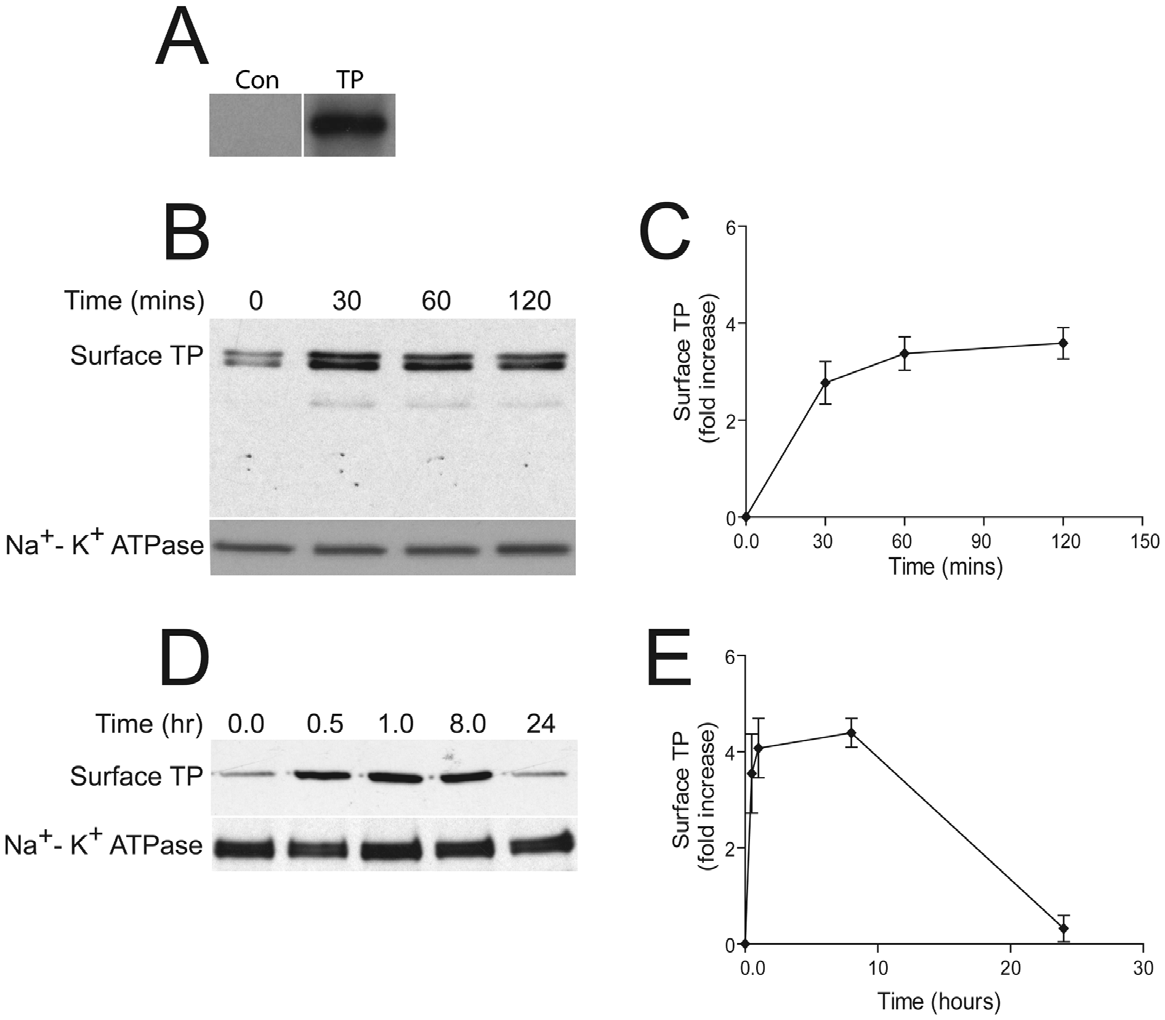
Effects of hydrogen peroxide exposure on cell-surface expression of TPβ. Panel A: HA-TPβ is detected only in transfected HEK293 cells. Cell-surface TPβ was labeled using a water-soluble biotin analogue and recovered with streptavidin beads. Steptavidin-bound proteins were analysed by immunoblotting using anti-HA antibodies. Lane 1 (Con) is the nontransfected negative control and lane 2 (TP) is transfected cells. Note the blot is from the same membrane but lanes have been rearranged for presentation only, and the greater density of the band here compared to panels B and C is due to longer exposure to enhance the negative control. Panels B-E: HEK293 cells stably expressing TPβ were incubated in DMEM, stimulated with 10 µM (final) hydrogen peroxide and then incubated for the times indicated. Cell-surface TPβ was detected as for panel A. Equal loading was confirmed by re-probing of membranes with antibodies against the Na^+^-K^+^ ATPase. Cell-surface levels were monitored over two hours (Panel B) or up to 24 hours (Panel D). Note doublet on panel B is due to a loading artifact from high volume samples and is seen only in some blots and does not indicate the presence of an additional form of TPβ. Panels C and E represent quantified data for panels B and D respectively where the values are means ± SEM of three independent experiments. Note that these graphs report ‘fold increase’ in surface TP expression; 0 represents no change from resting state expression. These data show that cell-surface HA-TPβ increases following H_2_O_2_ exposure ∼3.5 fold-increase after 2 hours. The extended time course in panels D and E demonstrates the H_2_O_2_-induced augmentation of cell-surface is sustained for at least 8 hours declining to resting values by 24 hours.

Cell surface expression of HA-tagged TPβ (HA-TPβ) was quantitated in HEK293 cells stably expressing HA-TPβ by surface biotinylation using a membrane impermeable biotin reagent followed by Western blotting to quantitate levels of biotin-derivatized receptor (Figure 1). In untransfected cells no HA-reactive material was detected by Western analysis of biotinylated material, but in contrast a clear band corresponding to the predicted molecular weight at ∼65 kDa of biotinyl-HA-TPβ was detected in transiently-transfected HEK293 cells (Figure 1A). This is consistent with the reported apparent molecular weight of TPβ, and also with our previous studies in COS cells [10]. When HEK293 cells were challenged with a single low level dose of H_2_O_2_ (10 µM final), initially comparatively low cell-surface expression of HA-TPβ was detected (Figure 1B and C). Strikingly, HA-TPβ levels increased at the cell-surface during the following 60 minutes (3.4±0.34 fold increase, n = 3) and remained elevated at two hours post-H_2_O_2_ treatment (3.6±0.32 fold increase, n = 3). To determine for how long increased surface expression of TPβ persisted an extended kinetic analysis of these cells, up to 24 hours post H_2_O_2_ challenge, was performed. These data indicated that the increase in cell-surface HA-TPβ was sustained for at least eight hours (4.3±0.3 fold increase, n = 3) following H_2_O_2_ treatment and declined to steady state levels within 24 hours (Figure 1D and E).

**Figure 2:**
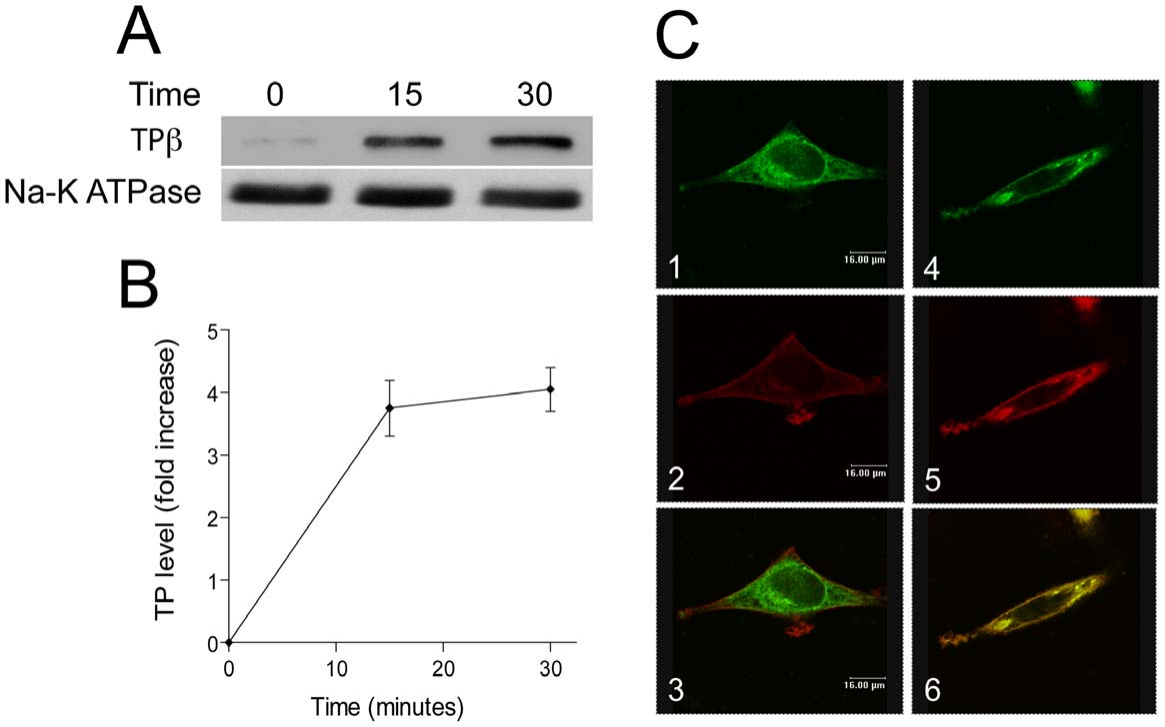
Effects of hydrogen peroxide on surface expression of endogenously expressed TP in PC3 prostate carcinoma cells. Panel A: TPβ detection on the surface of PC3 cells. Endogenous TPβ was labeled using a water-soluble biotin analogue and recovered with streptavidin beads at the cell surface following challenge with 10 µM hydrogen peroxide. Streptavidin-bound proteins were analysed by immunoblotting. Panel B: Quantitation of delivery of TPβ to the cell surface from two separate experiments, demonstrating rapid increase of streptavidin-accessible molecules. Panel C: Confocal imaging of TP translocation to the cell surface. Panels 1 to 3 are resting cells and 4 to 6 following hydrogen peroxide challenge at 40 minutes. Panels 1 and 4 are stained for TP, 2 and 5 for TRITC-phalloidin to visualize cortical actin, and 3 and 6, are merged images. Scale bar is 16 µm.

PC3 cells express a large intracellular pool of thromboxane receptor and both the TPα and TPβ isoforms are endogenously expressed, allowing examination of the effects of H_2_O_2_ on endogenous receptor and comparisons to HA-tagged TPβ expressed in HEK293 cells [6]. Exposure of PC3 cells to H_2_O_2_ resulted in an increase of endogenous TPβ at the cell-surface over 30 minutes, with strikingly similar kinetics to the behavior of HA-tagged TPβ expressed in HEK293 cells (Figure 2). These observations were confirmed by quantitation of the levels of biotin-captured TPβ and also by demonstration of translocation to the surface of TPβ, using confocal microscopy (Figure 2).

**Figure 3:**
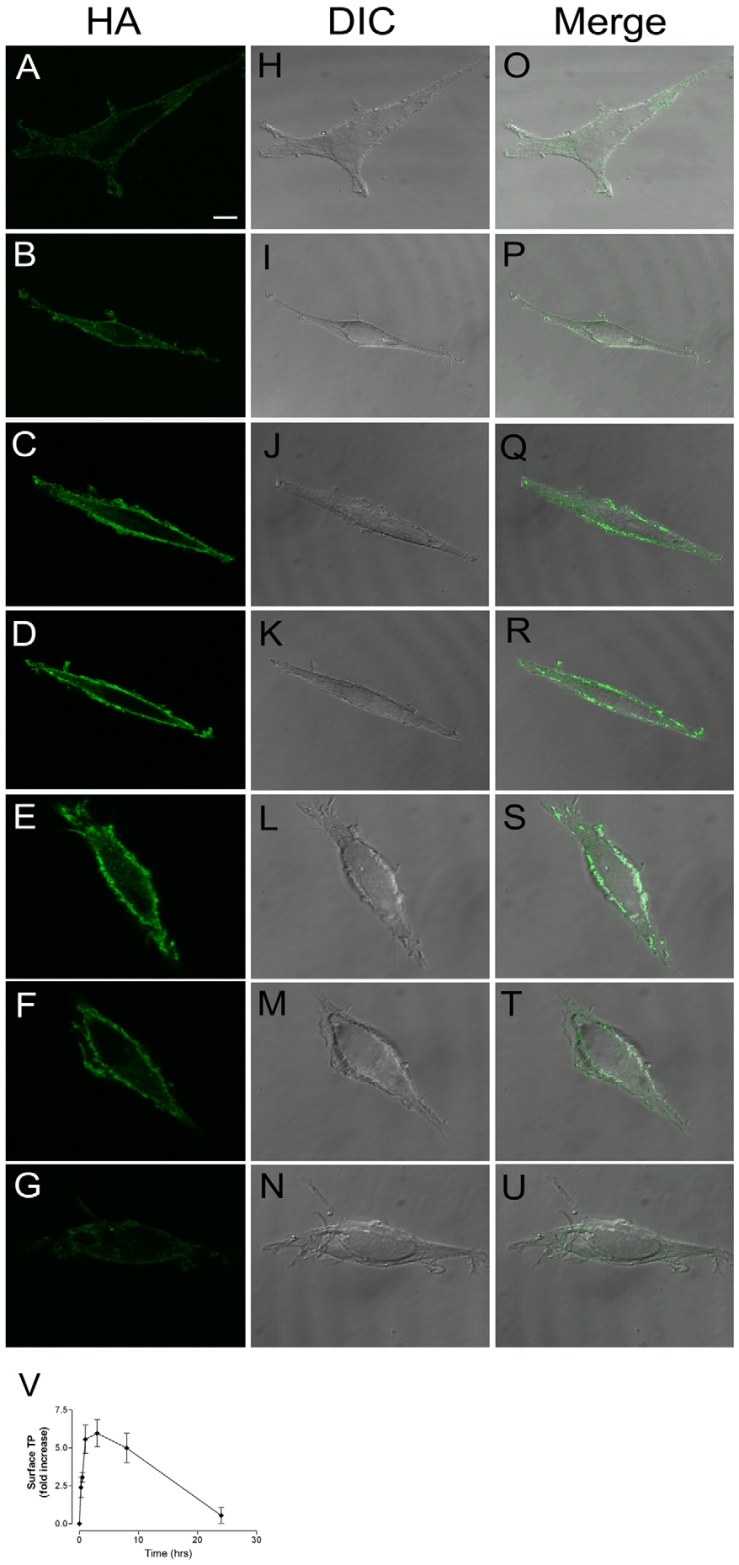
Confocal microscopy demonstrates increased cell-surface expression of HA-TPβ after hydrogen peroxide stimulation. HEK293 cells stably expressing HA-TPβ were visualized on glass-coverslips by staining with an anti-HA murine monoclonal and FITC-labelled goat anti-mouse antibodies under non-permeabilizing conditions. Indirect immunofluorescence images were collected using a Leica SP2 upright confocal microscope at times 0, 15, 30, 60 minutes and 3, 8 and 24 hours post a single challenge with hydrogen peroxide (10 µM) treatment (panels A–G). DIC images were also collected (H–N) and processed in Leica confocal software Lite to generate overlay images (O–U). Panel V represents the quantified confocal data (means ± SEM, n = 5). Fluorescence was quantitated in ImageJ 1.34s (NIH, USA) and normalized to t = 0. These data demonstrate cell-surface HA-TPβ increases ∼5-fold following hydrogen peroxide stimulation and predominates for approximately 8 hours, returning to non-stimulated levels 24 hours post-stimulation. Scale bar  = 5 µm.

Using confocal microscopy and indirect immunofluorescence of nonpermeabilized cells, a kinetic analysis of surface fluorescence demonstrated rapid and persistent increased detection of the HA-epitope in HEK293 cells expressing HA-TPβ and following exposure to H_2_O_2_ (Figure 3A–U). The HA-epitope, which is located at the N-terminus of the TPβ construct is exposed to the surface and hence accessible to antibody added to nonpermeabilized HEK293 cells. Quantification of the FITC-fluorescence intensity associated with the increased surface expression of TPβ (Fig. 3 panel V) confirmed that HA-TPβ increased at the plasma membrane within 30 minutes of exposure to oxidative stress (3.1±0.7 fold increase, n = 5). Peak surface expression was observed within 60 minutes (5.5±0.9 fold increase n = 5), which slowly declined to resting state levels within 24 hours. These data correlate very well with the biotinylation studies and provided an independent method for determination of cell surface expression of HA-TPβ.

### Increased TPβ expression at the cell surface is accompanied by concomitant loss from internal pools

**Figure 4:**
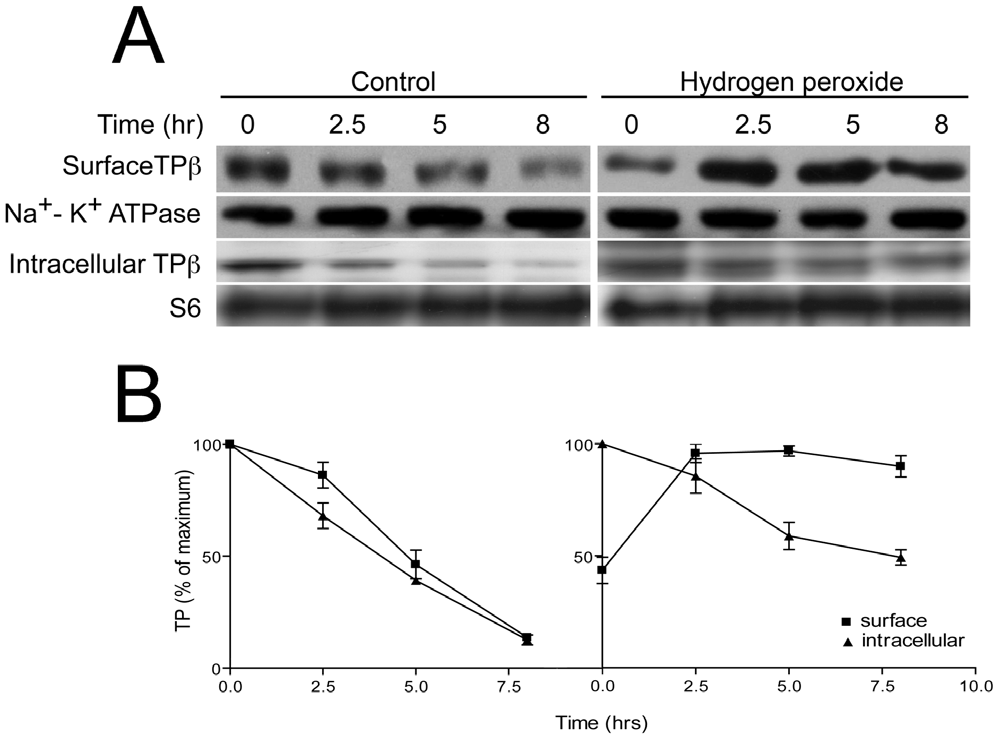
Kinetic analysis of intracellular and cell-surface HA-TPβ levels following hydrogen peroxide exposure. Cycloheximide (30 µM) was added to HEK293 cells stably expressing HA-TPβ and kinetic studies performed in the absence and presence (Panel A) of hydrogen peroxide (10 µM). At times 0, 2.5, 5 and 8 hours following addition of cycloheximide and hydrogen peroxide the cultures were placed on ice and cell-surface proteins labelled with a water-soluble biotin analogue. Labelled cell-surface HA-TPβ was recovered by incubating with streptavidin beads and the remaining intracellular fraction precipitated using trichloroacetic acid. Both fractions were analyzed using anti-HA murine monoclonal and HRP-conjugated secondary antibodies. Equal loading was confirmed by reprobing membranes with anti-Na^+^-K^+^ ATPase antibody (surface) and anti-ribosomal S6 antibodies (total lysate). Re-probing the membrane fraction with S6 antibodies indicated the absence of any contamination by intracellular proteins, i.e. from cell lysis (data not shown). Panel B represents the quantified data expressed as percentage change of intracellular and cell-surface HA-TPβ expression in the absence and presence of hydrogen peroxide, with data normalized to t = 0. In the absence of hydrogen peroxide, both cell-surface and intracellular HA-TPβ decline over 8 hours, but the hydrogen peroxide treated cultures demonstrate sustained HA-TPβ levels. The data are representative of the means ± SE of three independent experiments.

HEK293 cells stably expressing HA-TPβ were pre-dosed for 30 minutes with cycloheximide, or vehicle alone (final concentration 0.0004% (v/v) ethanol) and cell-surface HA-TPβ was labelled using a water-soluble biotin analogue and captured with streptavidin beads as before. The remaining intracellular fraction was precipitated using trichloroacetic acid (Fig. 4A and B). Na^+^-K^+^ ATPase and ribosomal S6 protein were used as loading controls for biotin accessible and inaccessible fractions respectively. In addition the biotinylated fractions were reprobed with the anti-S6 antibody; no reactivity was detected, ruling out non-specific cell lysis during the procedure (data not shown). In the absence of H_2_O_2_, cell-surface HA-TPβ decreased to ∼20% (n = 3) of resting levels after 8 hours (Figure 4A). This observation contrasts to the substantial H_2_O_2_-induced cell-surface HA-TPβ increase, which, in the presence of cycloheximide, was sustained over 8 hours (Figure 4B). Similarly, intracellular HA-TPβ was more rapidly degraded in the absence of H_2_O_2_, decreasing to ∼20% (n = 3) after eight hours, compared to nearly 50% (n = 3) in the H_2_O_2_ treated cells.

### Increased TPβ surface expression is sensitive to brefeldin A

**Figure 5:**
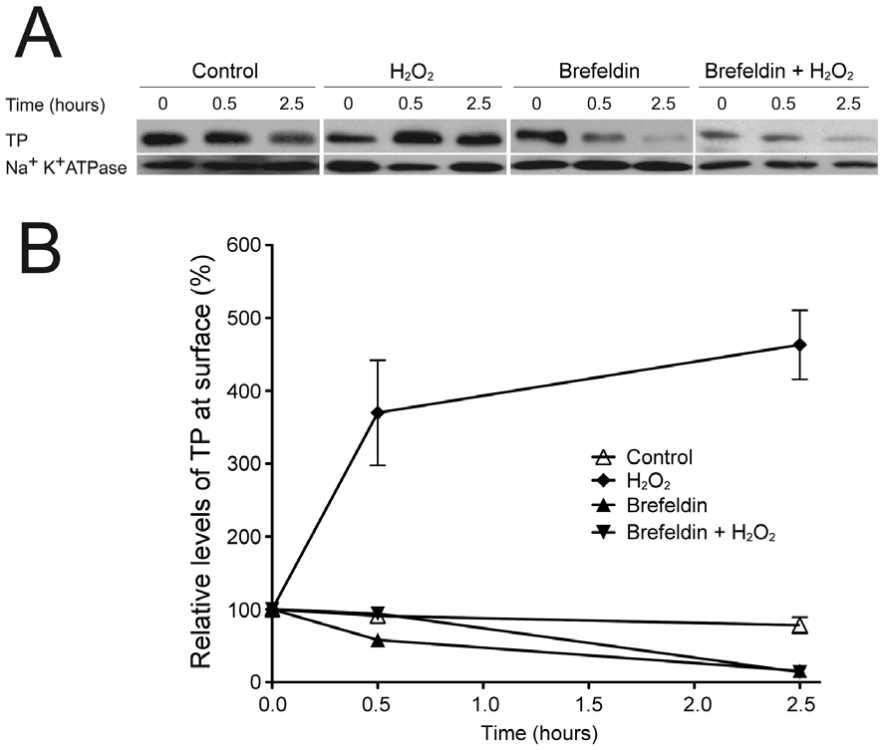
Increased surface expression of TPβ is sensitive to brefeldin A. Kinetic analysis of HEK293 cells stably expressing HA-TPβ were performed in the absence and presence of brefeldin A. Cultures were treated with cycloheximide (30 µM) and brefeldin A (10 µM) prior to the addition of hydrogen peroxide (10 µM). At times 0, 0.5 and 2.5 hours post hydrogen peroxide exposure, cell-surface proteins were labeled with a water-soluble biotin analogue for 30 minutes on ice and recovered on streptavidin beads. TPβ was analysed by immunoblotting with anti-HA monoclonal antibodies. Panels are representative data from three independent experiments where loading was controlled by reprobing membranes with anti-Na^+^-K^+^ ATPase antibodies.

HEK293 cells expressing HA-TPβ were treated with cycloheximide and H_2_O_2_, and the effect of brefeldin A on surface delivery assessed by biotinylation and Western analysis (Figure 5). In the absence of brefeldin A, and consistent with our previous studies, H_2_O_2_ induced increased expression of HA-TPβ at the cell surface. By contrast, pretreatment of cells with brefeldin A abolished this cell-surface HA-TPβ delivery. This suggests that transit through the Golgi complex is required for surface delivery, and is consistent with our earlier data that demonstrated translocation of the ER pool to the Golgi complex rapidly following H_2_O_2_ stimulation. Taken together with the data above, these observations indicate that H_2_O_2_ leads to translocation of TPβ into the Golgi apparatus, and that this pool is then routed to the cell surface.

### Involvement of the proteasome in cell-surface expression of TPβ

**Figure 6:**
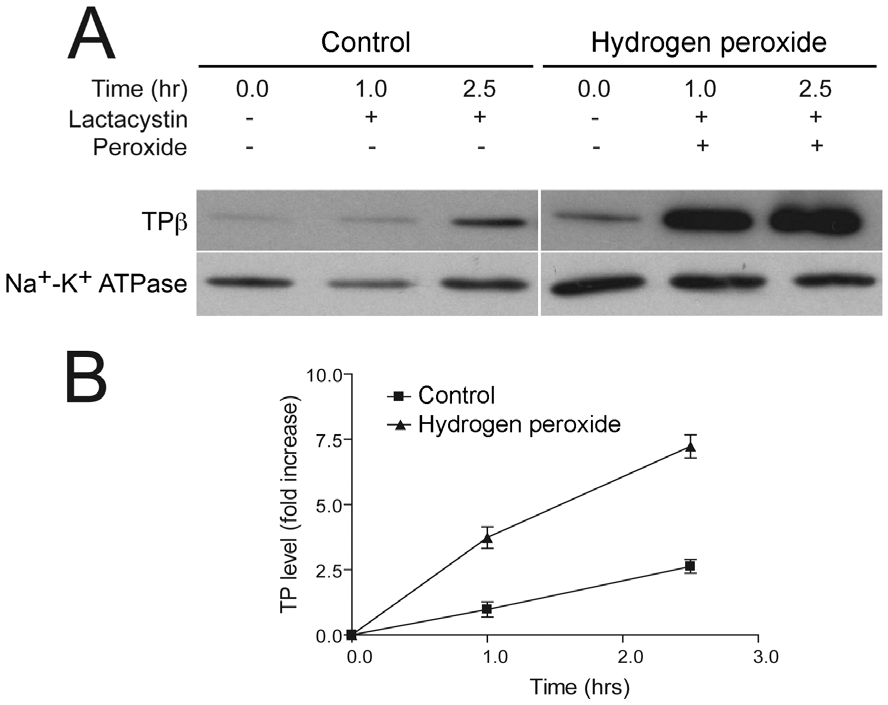
Turnover of TPβ and control of cell-surface expression requires proteasomal function. Inhibition of the proteasome in HEK293 cells stably expressing HA-TPβ was achieved by incubating the cultures with lactacystin (1 µM) and cycloheximide (30 µM) both in the absence and presence of H_2_O_2_ (Panel A). At times 0, 1 and 2.5 hours, cell-surface HA-TPβ was labelled with a water-soluble biotin analogue and bound to streptavidin beads. Recovered HA-TPβ was probed by Western blotting with anti-HA antibodies. Quantified data (Panel B), normalized to control cells suggest that the proteasome plays a role in the negative regulation of cell-surface HA-TPβ during oxidative stress. Data are representative of the means ± SEM of three independent experiments where the presence of hydrogen peroxide caused a significant increase in HA-TPβ (P<0.05) at 2.5 hours (*) compared to the non-H_2_O_2_ group.

HEK293 cells stably expressing HA-TPβ were treated with lactacystin, an irreversible inhibitor of the proteasome, in the presence of cycloheximide. Cell-surface HA-TPβ was assayed at different time periods with a membrane impermeable biotin analogue both in the absence and presence of H_2_O_2_ and recovered with streptavidin beads (Figure 6). The duration of the experiment was limited to 2.5 hours (more prolonged periods manifested toxic effects from the combined drug treatment). TPβ recovery was normalised using Na^+^-K^+^ ATPase antibodies as loading control. Interestingly, after 2.5 hours, lactacystin treatment alone produced a modest ∼2-fold increase in HA-TPβ at the cell-surface, reflecting considerable proteasomal-dependent degradation of the receptor even in the resting state, but consistent with turnover of intracellular pools by ER-associated degradation (ERAD). When cells were challenged with H_2_O_2_ together with lactacystin, a dramatically increased level of TPβ was detected at the cell surface, approaching eight-fold at 2.5 hours. As this increase is significantly greater than that observed in the absence of lactacystin these data suggest that the proteasome contributes to turnover of TPβ, and potentially turnover from the surface pool.

### H_2_O_2_ exposure induces translocation of functional thromboxane receptor to the plasma membrane

**Figure 7:**
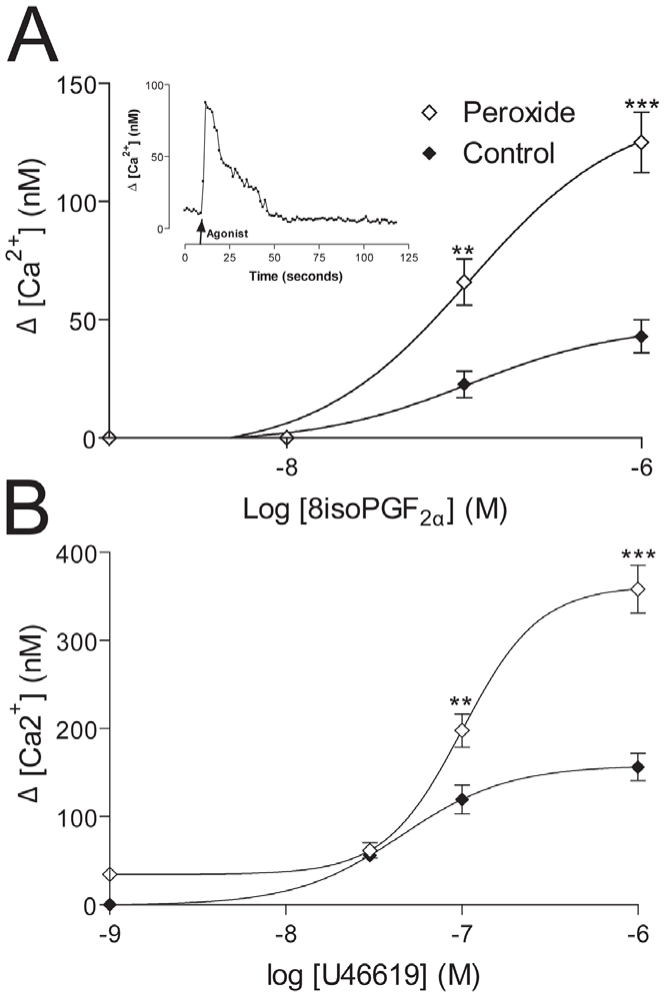
Concentration-response effect of thromboxane mimetic U44619 and 8-iso PGF_2α_ on calcium flux in HEK293 cells stably expressing HA-TPβ. HEK293 cells were loaded with FuraRed (20 µM) for 30 minutes at 37°C. Cell visualization was performed using a Zeiss Axiovert 200 microscope and data processed using Volocity software (Improvision). Cytosolic calcium levels in response to stimulation were determined in the absence or presence of hydrogen peroxide (10 µM) and calculated as described under methods. Figure A inset represents a single trace following the addition of 8-iso PGF_2α_ (nM). Following addition of agonist at the point indicated in the figure, a rapid increase in calcium concentration is observed (∼100 nM) which is sustained for ∼60 seconds. Similar response curves were generated for 8-iso PGF_2α_ (Panel A) and U46619 (Panel B). Data represent the mean ± SEM (n = 7), ** P<0.01, *** P<0.001.

To determine of the increased pool of TPβ was functional and capable of signal transduction, we analysed calcium mobilization in HEK293 cells stably expressing HA-TPβ. We have previously shown that in COS-7 cells H_2_O_2_ results in an increase in the number of thromboxane-binding sites in whole cell extracts, but did not demonstrate the ability to engage in downstream signaling [10]. Cells were preloaded with FuraRed in the presence of the mild detergent pluronic F127. Preincubation of non-transfected HEK293 cells with the TPβ antagonist SQ29548 or the TPβ agonist U46619 confirmed there was no significant background Ca^2+^ flux (data not shown), consistent with a low level of expression of TPβ in these cells. The effects of U46619, a thromboxane A_2_ mimetic, and 8-iso PGF_2α_ on calcium mobilization at agonist concentrations between 1 nM and 1 µM were examined in the absence and presence of H_2_O_2_ in the HEK293 cells stably expressing HA-TPβ (Fig. 7). Following addition of agonist a significant and transient rise (∼75 seconds) in intracellular calcium was observed (Fig. 7 inset). The increase in intracellular calcium was far greater than in control cells in the absence of hydrogen peroxide (Supplementary Figure S1). Both U46619 and 8-iso PGF_2α_ at concentrations between 10 nM and 1 µM produced an increase in intracellular calcium concentration in non-H_2_O_2_-treated cells. However, in the presence of H_2_O_2_, this effect was markedly potentiated, suggesting that the increase in TPβ levels facilitated greater Ca^2+^ mobilization (Fig. 7A and B).

**Figure 8:**
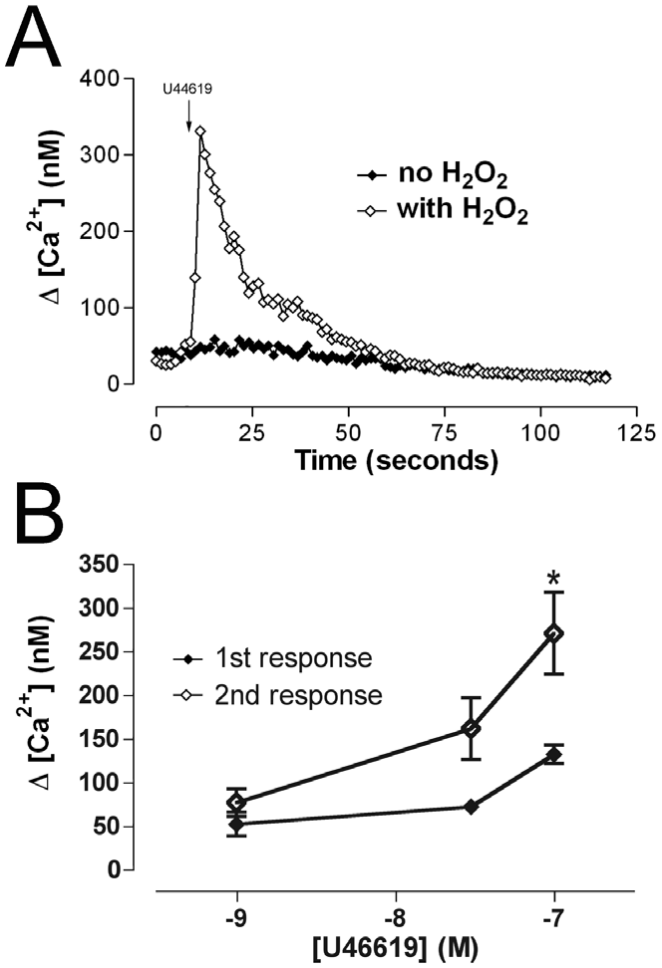
Hydrogen peroxide exposure relieves receptor desensitization and leads to potentiation of calcium mobilization following repeat challenge by U46619. HEK293 cells stably expressing HA-TPβ were loaded with FuraRed (20 µM) for 30 minutes at 37°C in the presence of pluronic F127. Cultures were given an initial challenge of U46619 (1, 30 and 100 nM) and visualized under a Zeiss Axiovert 200 microscope. Cytosolic calcium concentration was calculated as described in methods. Following the first challenge, cells were washed three times in recording buffer and incubated for a further 30 minutes at 37°C. A second challenge of U46619 was carried out as previously. Panel A is representative single traces demonstrating the effect of receptor desensitization in the presence and absence of hydrogen peroxide. Following an initial challenge of U46619 the second dose of ligand (100 nM) was added at the point indicated in the figure. In the absence of hydrogen peroxide an additional calcium transient was not detected, but by contrast, a calcium transient was observed in H_2_O_2_-treated cells. Panel B shows concentration-response data for the repeated U46619 challenge in the presence of hydrogen peroxide (10 µM). These observations were found to be significant (P<0.05) at 100 nM (*), the data representing the mean ± SEM, n = 7.

Further experiments were carried out to investigate the effects of a second U46619 challenge 30 minutes following exposure to the initial stimulus. In non-H_2_O_2_ treated cells, exposure to a second U46619 challenge failed to elicit an observable calcium response consistent with normal downregulation of responsiveness (Fig. 8A). Unexpectedly, in the presence of H_2_O_2_ a second administration of U46619 produced a highly unusual and marked potentiation of calcium mobilization (Fig. 8A and B). It should be noted that the repeat stimulation experiment was performed after translocation of TPβ to the cell surface is complete, i.e. 60 minutes (Figures 1 and 3), and therefore the increased second response cannot be ascribed alone to a further increase in the surface pool. These observations suggest that H_2_O_2_ exposure results in an alteration to the TPβ microenvironment, such that the normal downregulation in Ca^2+^ mobilization has been avoided, and which could potentially be due to a change to the turnover kinetics of TPβ. While it is possible that hydrogen peroxide affects calcium mobilization through a mechanism independent of the TPβ receptor, and that hydrogen peroxide alone is capable of inducing calcium mobilization [17], [18], [19], these earlier investigations used hydrogen peroxide concentrations very much higher than the current study and we observed that treatment with 10 µM hydrogen peroxide had no observable effect on calcium mobilization in the absence of TPβ agonist.

### H_2_O_2_ exposure attenuates ligand-induced internalization of the thromboxane receptor

**Figure 9:**
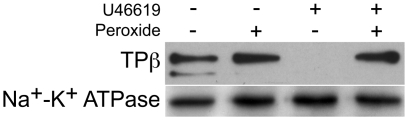
Effect of hydrogen peroxide on ligand-induced internalization of TPβ. HA-TPβ internalization in response to U46619 was studied in HEK293 cells stably expressing TPβ, both in the absence and presence of H_2_O_2_. Cultures were treated with H_2_O_2_ (10 µM) for 40 minutes prior to the addition of U46619 (600 nM) and incubated for a further 90 minutes at 37°C. Cell-surface proteins were labelled with a water-soluble biotin analogue followed by adsorption onto streptavidin beads. HA-TPβ was detected with anti-HA antibodies following electrophoresis and transfer to membrane and loading equivalence assessed by reprobing membranes with Na^+^-K^+^ ATPase antibodies. Data are representative of three independent experiments.

Ligand-induced internalization studies were performed both in the presence and absence of hydrogen peroxide in HEK293 cells stably expressing HA-TPβ. Determination of the surface expression of TPβ was once again performed by surface biotinylation and Western blotting, where protein synthesis was blocked by cycloheximide. Addition of H_2_O_2_ was found to lead to an increase in detectable surface TPβ after two hours as before (Figure 9). By contrast, addition of U46619 was found to decrease the detectable surface TPβ, suggesting that in the resting state the receptor is internalized following ligand stimulation. More interestingly, this ligand-dependent internalization was abolished in the presence of hydrogen peroxide. This is consistent with the increased Ca^2+^ mobilization observed on repeat ligand challenge and also with the long half-life of receptor at the cell surface following H_2_O_2_ challenge (Figures 1, 2 and 3).

### Specificity in response to H_2_O_2_ challenge amongst prostanoid receptors

**Figure 10:**
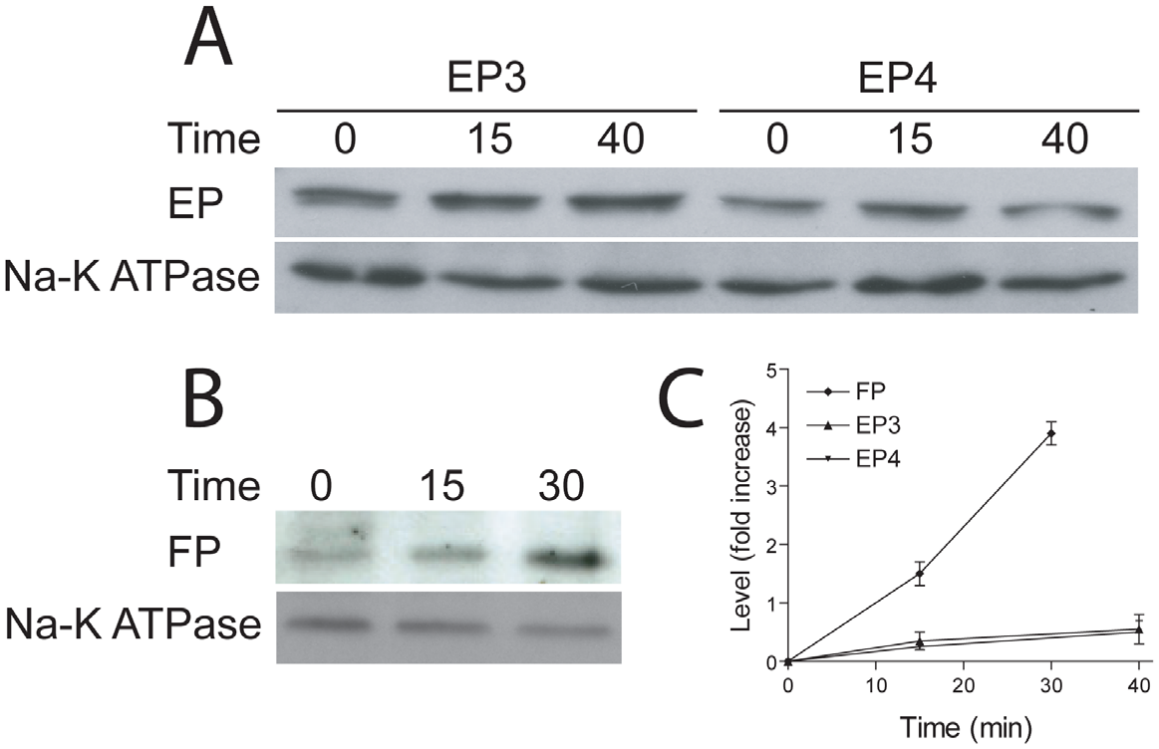
Hydrogen peroxide treatment augments surface levels of some, but not all, prostanoid receptors. Panels A and B: HA-tagged EP3, EP4 and FP transiently transfected into HEK293 cells, and surface levels quantitated by derivatizing with a water-soluble biotin analogue and recovered with streptavidin beads following challenge with 10 µM hydrogen peroxide. The Na^+^-K^+^ ATPase is used in all cases as a loading control. Panel C: Quantitation from two similar experiments for surface levels of EP2, EP3 and FP. There is a clear increase in FP levels, but not for EP2 or EP3.

To examine if the alterations in stability and location described here and previously [10] for both isoforms of TP following H_2_O_2_ exposure were restricted to TP or extend to additional members of the prostanoid receptor family, we investigated the stability of EP isoforms 3 and 4 and also FP. We constructed HA-tagged expression vectors for these receptors and expressed then in HEK293 cells as before, and detected HA-reactive bands with migrations consistent with the predicted molecular weights in whole cell lysates (Figure 10 and data not shown). Analysis of the influence of H_2_O_2_ on membrane-impermeable biotin-accessible levels of each receptor revealed a differential response to oxidative stimulus. Specifically, surface levels of neither EP3 nor EP4 were affected by exposure to H_2_O_2_, while by contrast levels of FP accessible to the surface probe were significantly and rapidly increased (Figure 10). We did not investigate the mechanism underpinning this increase or the persistence of the response further, but we consider it likely that the UPR mediates the increase in FP levels. Regardless of mechanism, these data clearly indicate that the expression levels of members of the prostanoid receptor family display differential sensitivity to cellular hydrogen peroxide exposure, providing potentially complex responses to oxidative stresses in vivo.

## Discussion

Thromboxane (TXA_2_) is a potent signaling molecule under normal physiological conditions, and is the product of a distinct range of cell types [20]. Tight regulatory mechanisms exist to attenuate signaling via TXA_2_, and most notably TXA_2_ is rendered biologically inactive within 30 seconds of production [21]. In certain pathophysiological states, however, the effects of TXA_2_ may become detrimental. Over-production of TXA_2_ in the vasculature results in local vasoconstriction and platelet aggregation; the resulting impedance to blood flow can lead to life-threatening conditions such as myocardial infarction [22]. Concomitant oxidative stress also stimulates synthesis of another thromboxane receptor agonist, 8-iso prostaglandin F_2α_, one of the more abundant isoprostanes formed in vivo [23] and present in atherosclerotic coronary arteries [24].

We previously demonstrated that the vasoconstrictive action of 8-iso prostaglandin F_2α_, is mediated via the TP receptor [25] and that the response to this isoprostane is enhanced by oxidative stress [26]. We hypothesized that this additional responsiveness to the isoprostane was due to an increased TPβ receptor reserve. More recently we provided empirical evidence to support this hypothesis by demonstrating that oxidative stress promoted TPβ receptor translocation from the ER to the Golgi complex, which was accompanied by stabilization [10]. The present study extends that work by demonstrating that simulating oxidative stress in isolated cells by treatment with hydrogen peroxide causes an enhanced and prolonged increase in the response of TPβ receptor to ligand binding, and that this is most likely a result of an increase in cell surface TPβ receptor population mediated via cellular alterations of the TPβ receptor at multiple levels. We also provide evidence that a similar mechanism may exist for the FP prostanoid receptor, but that this does not extend to the EP3 and EP4 receptors, suggesting differential regulatory mechanisms within the overall prostanoid receptor family.

Initially we considered the surface pool of the TPβ receptor. Biotinylation and immunofluorescence microscopy demonstrated rapid and prolonged relocation of TPβ from the ER to the cell surface, maximal within 1 hour and persisting for 8 hours. Resting levels were restored after 24 hours. Therefore oxidative stress induced a rapid sustained upregulation of HA-TPβ at the cell-surface. Hydrogen peroxide has a short half-life in cell culture medium (up to 20 minutes) [27], [28], [29], hence we conclude that a single, brief challenge with hydrogen peroxide is able to trigger a prolonged change in cell surface expression of HA-TPβ. Secondly, by examining the effects of H_2_O_2_ on PC3 cells, which endogenously express a large intracellular pool of both the TPα and TPβ isoforms [6], it was found that the endogenous TP receptor in PC3 cells accumulated at the cell surface upon exposure to H_2_O_2_ with strikingly similar kinetics to HA-TPβ in transiently-transfected HEK293 cells. While expression of Na^+^-K^+^ ATPase, used as the loading control in these experiments, can itself be modulated by ROS [30], there was no evidence for this in our experiments, and the concentrations of H_2_O_2_ used here are very much lower than those required to elicit such an effect on the Na^+^-K^+^ ATPase.

Next we considered intracellular transport and turnover of TPβ at several levels. Firstly, both internal and surface pools of TPβ normally turn over with a half life of ∼4 hours in HEK293 cells, similar to ∼2.5 hours in COS-7 cells [10]. In cells exposed to H_2_O_2_ there is significant stabilization of TPβ receptor and the intracellular half-life increases to >8 hours. Again, the presence of increased receptor at the cell surface is detected with a concomitant decrease in the intracellular fraction. Secondly, translocation of TPβ to the surface was prevented by brefeldin A, an antibiotic with specific effects at the Golgi complex. [31], [32]. As TPβ is translocated from endoplasmic reticulum-localized pools to structures colocalizing with Golgin-97 in COS-7 cells [10], together these data indicate that the ER pool of TPβ receptor is stabilized and delivered to the cell surface following H_2_O_2_ stimulation by anteriograde vesicular transport. Thirdly, in the presence of lactacystin, a modest ∼two-fold increase in TPβ receptor at the cell-surface suggests proteasomal involvement in ER-derived TPβ degradation [33]. While oxidative stress inactivates the proteasome a very prolonged incubation with hydrogen peroxide is required [34], and hence the single pulse used here is not expected to disrupt proteasome function. As quality control mechanisms prevent ER exit these data may indicate that additional receptor molecules can achieve the native state if degradation is prevented and the period available for folding extended. The almost eight-fold increase in TPβ receptor at the surface in cells treated with both H_2_O_2_ and lactacystin suggests an important role for the proteasome in TPβ translocation, and possibly in turnover of surface pools. Therefore the proteasome degrades a significant population of TPβ and this accounts for ER retention and failure to reach the cell surface. Our investigations also show that, not only is there slower turnover of cell-surface TPβ following exposure to hydrogen peroxide, but also that this population escapes ERAD. Finally, expression of the FP receptor also rapidly increases at the cell surface following H_2_O_2_ exposure, most likely by a similar mechanism to TP, but that this does not extend to EP3 and EP4. Importantly, these data indicate both sequence-specific responses to H_2_O_2_, and also suggest that the physiological response of prostanoid receptors to oxidative stress exhibits unexpected specificity.

**Figure 11:**
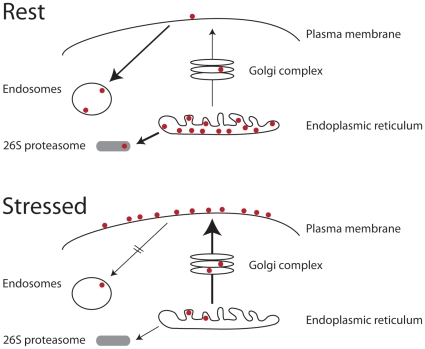
A model for intracellular transport of TP in resting and hydrogen peroxide-challenged cells. At rest (upper panel), most TP is retained in the ER and degraded by the 26S proteasome following dislocation to the cytosol. The small quantities of receptor that do exit the ER and enter into the secretory system are degraded and removed from the cell surface efficiently, presumably by endocytosis. Following exposure to hydrogen peroxide (lower panel), the steady state distribution is altered so that TP levels at the surface are greatly increased. This results from the combination of more efficient ER exit, due to increased chaperone concentrations, and an increased surface half-life. It is presumed that the latter is in part the result of a decrease in endocytosis of TP. TP molecules are shown as maroon dots, and the proteasome as a gray lozenge. Arrow thickness is proportional to flux between destinations.

A model for these changes to TPβ trafficking behavior via exposure to ROS is shown in Figure 11. It is important to note that TPα and TPβ have distinct internalization characteristics and that these differences are imparted by their unique cytoplasmic tails. Agonist-induced internalization of TPβ is dynamin- and β-arrestin-dependent, suggesting clathrin-mediated endocytosis [35], [36]. The Rab11 GTPase, which mediates endosomal recycling pathways, is also involved in control of cell-surface TPβ expression [37]. How H_2_O_2_ interacts with any of these mechanisms to effect TPβ stabilization at the cell-surface remains to be understood, and while clearly an important aspect of TPβ receptor function, is beyond the scope of this study.

Finally, we sought to investigate directly the capability of the surface TPβ pool as a signaling platform. TPβ receptor agonists U46619 and 8-iso PGF_2α_ produced an increase in intracellular calcium concentration in resting cells, and the effect markedly potentiated following H_2_O_2_ exposure. A second application of TPβ receptor agonist in the presence of H_2_O_2_ potentiated the first response, in contrast to control cells where a second response to agonist was absent. Taken together, these data clearly demonstrate that the H_2_O_2_-augmented cell-surface TPβ pool is functional and exquisitely capable of evoking increased ligand-responsiveness. Further, exposure to H_2_O_2_ abolished agonist-dependent TPβ receptor internalization, which could be achieved by decreased endocytosis, more efficient recycling or a combination of both. TPβ is down-regulated from the cell-surface following exposure to agonist [36] and persistence at the surface during oxidative stress could explain the calcium mobilization following the second agonist challenge. Clearly these data indicate altered engagement with the endocytic system, possibly through changes to the membrane microenvironment or expression of factors within the endocytic apparatus itself. Regardless of the precise mechanism, these data indicate a significant change to receptor fate and a further contribution to enhanced cellular prostanoid hypersensitivity.

In summary, a single, brief oxidative insult is sufficient to induce rapid and persistent translocation of TPβ receptor to the surface; the surface cohort is functional and hyper-responsive to agonist. Together these processes contribute to a sophisticated and dramatic mechanism for augmentation of prostanoid responsiveness following exposure to ROS. We believe this to be the first report of such complex effects on a G protein-coupled receptor and these findings have a significant implication for our understanding of receptor regulation. Specifically, an increase to the cell-surface TPβ population could dramatically alter physiological responsiveness to thromboxane receptor agonists.

## Supporting Information

Figure S1 Calcium flux in HEK293 cells stably expressing HA-TPβ in the absence of hydrogen peroxide. Figure represents a single trace following the addition of 8-iso PGF2α (nM). HEK293 cells were loaded with FuraRed (20 µM) for 30 minutes at 37°C. Cell visualization was performed using a Zeiss Axiovert 200 microscope and data processed using Volocity software (Improvision). Cytosolic calcium levels in response to stimulation were determined in the absence of hydrogen peroxide. Inset is the same data as in Figure 7.
(3.61 MB TIF)
