# Supplementary figures and images for: Regulation of Thromboxane Receptor Signaling at Multiple Levels by Oxidative Stress-Induced Stabilization, Relocation and Enhanced Responsiveness
Source: PLoS One. 2010 Sep 15;5(9):e12798. doi: 10.1371/journal.pone.0012798 (PMC2939892; doi:10.1371/journal.pone.0012798)

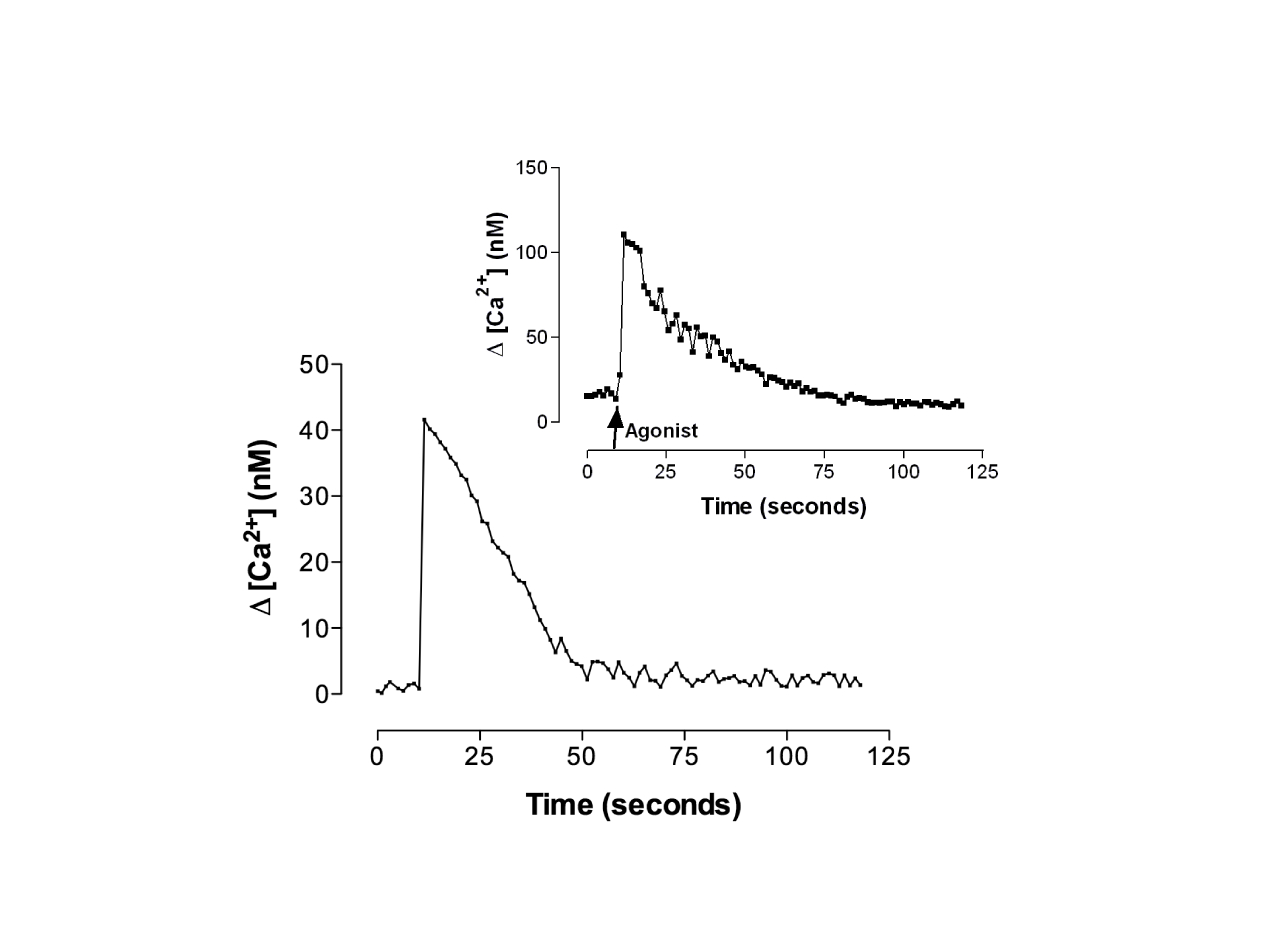

Supplement: Figure S1 — Calcium flux in HEK293 cells stably expressing HA-TPβ in the absence of hydrogen peroxide. Figure represents a single trace following the addition of 8-iso PGF2α (nM). HEK293 cells were loaded with FuraRed (20 µM) for 30 minutes at 37°C. Cell visualization was performed using a Zeiss Axiovert 200 microscope and data processed using Volocity software (Improvision). Cytosolic calcium levels in response to stimulation were determined in the absence of hydrogen peroxide. Inset is the same data as in Figure 7. (3.61 MB TIF) [file pone.0012798.s001.tif]
